# Evidence of Infection with Zoonotic Mosquito-Borne Flaviviruses in Saltwater Crocodiles (*Crocodylus porosus*) in Northern Australia

**DOI:** 10.3390/v14051106

**Published:** 2022-05-21

**Authors:** Gervais Habarugira, Jasmin Moran, Jessica J. Harrison, Sally R. Isberg, Jody Hobson-Peters, Roy A. Hall, Helle Bielefeldt-Ohmann

**Affiliations:** 1 School of Veterinary Science, The University of Queensland, Gatton, QLD 4343, Australia; g.habarugira@uq.net.au; 2 Centre for Crocodile Research, Noonamah, NT 0837, Australia; research@crocresearch.com.au (J.M.); sally@crocresearch.com.au (S.R.I.); 3 School of Chemistry and Molecular Biosciences, The University of Queensland, St. Lucia, QLD 4072, Australia; j.harrison1@uq.edu.au (J.J.H.); j.peters2@uq.edu.au (J.H.-P.); roy.hall@uq.edu.au (R.A.H.); 4 Australian Infectious Diseases Research Centre, The University of Queensland, St Lucia, QLD 4072, Australia

**Keywords:** flavivirus, crocodile, mosquito-borne, neutralising antibody

## Abstract

The risk of flavivirus infections among the crocodilian species was not recognised until West Nile virus (WNV) was introduced into the Americas. The first outbreaks caused death and substantial economic losses in the alligator farming industry. Several other WNV disease episodes have been reported in crocodilians in other parts of the world, including Australia and Africa. Considering that WNV shares vectors with other flaviviruses, crocodilians are highly likely to also be exposed to flaviviruses other than WNV. A serological survey for flaviviral infections was conducted on saltwater crocodiles (*Crocodylus porosus*) at farms in the Northern Territory, Australia. Five hundred serum samples, collected from three crocodile farms, were screened using a pan-flavivirus-specific blocking ELISA. The screening revealed that 26% (*n* = 130/500) of the animals had antibodies to flaviviruses. Of these, 31.5% had neutralising antibodies to WNV_KUN_ (Kunjin strain), while 1.5% had neutralising antibodies to another important flavivirus pathogen, Murray Valley encephalitis virus (MVEV). Of the other flaviviruses tested for, Fitzroy River virus (FRV) was the most frequent (58.5%) in which virus neutralising antibodies were detected. Our data indicate that farmed crocodiles in the Northern Territory are exposed to a range of potentially zoonotic flaviviruses, in addition to WNV_KUN_. While these flaviviruses do not cause any known diseases in crocodiles, there is a need to investigate whether infected saltwater crocodiles can develop a viremia to sustain the transmission cycle or farmed crocodilians can be used as sentinels to monitor the dynamics of arboviral infections in tropical areas.

## 1. Introduction

Flaviviruses are members of the family Flaviviridae, which are mostly mosquito-borne viruses with a worldwide distribution. The family comprises four main genera, including flavivirus, pestivirus, pegivirus, and hepacivirus [1]. Flaviviruses are positive-sense, single-stranded RNA (ssRNA(+)),enveloped and monopartite viruses, with an unsegmented linear viral genome of approximately 9.4–13 kb, enclosed in an icosahedral nucleocapsid of 50nm in diameter [2]. Flaviviruses are generally transmitted by arthropods, including mosquitoes and ticks, and the final hosts are usually mammals, birds, and reptilians [1,3]. Some flaviviruses are pathogenic and can cause severe diseases in humans and animals [4]. Serologically, mosquito-borne flaviviruses are classified in multiple serocomplexes. The Japanese encephalitis virus (JEV) serocomplex comprises the JEV, West Nile (WNV), Usutu (USUV), Murray Valley encephalitis (MVEV), Yaounde, Alfuy (ALFV), and Saint Louis encephalitis (SLEV) viruses. The yellow fever virus (YFV) serocomplex comprises the YFV, Fitzroy River (FRV), Sepik (SEPV), Edge Hill (EHV), Bouboui, Banzi, Jugra, Saboya, and Potiskum viruses. YFV and SEPV are the two most closely related in the YFV serogroup. The dengue virus (DENV) serogroup comprises DENV1, DENV2, DENV3, and DENV4. The Spondweni virus complex comprises the Spondweni and Zika (Brazilian and African strains) viruses. The Kokobera virus (KOKV) serocomplex group comprises the KOKV, Stratford (STRV), and New Mapoon (NMV) viruses. The KOKV serocomplex group is closely related to the Aroa virus complex viruses that comprise the Iguape, Naranjal, and Bussuquara viruses. The Ntaya virus serocomplex comprises the Bagaza, Ntaya, Tembusu, Ilheus, and Rocio viruses.

In Australia, the most clinically significant flaviviruses that infect and cause disease in humans are MVEV, WNV_KUN_ (Kunjin strain), DENV, and JEV [5,6,7]. Of these, most cases of DENV and JEV are imported. DENV is no longer endemic to Australia, but travel-associated outbreaks occur regularly, mainly in Northern Queensland [8]. There were JEV outbreaks in the Cape York Peninsula and Torres Strait Islands in 1995 and 1998, respectively [9,10,11]. Recently, JEV outbreaks have been reported in Victoria, Queensland, New South Wales, and South Australia. Currently, 34 human cases, of which three were fatal, have been recorded. Additionally, JEV cases have been reported in 70 piggeries across Australia [12,13,14]. However, other flaviviruses, such as KOKV, ALFV, STRV, and EHV, have been reported to be associated with a few human disease cases, usually with non-specific fevers [15,16].

MVEV is the leading cause of viral encephalitis in Australia and endemic in parts of Western Australia and the Northern Territory. The virus is maintained between aquatic birds and the vector mosquito *Culex annulirostris* [17,18]. There have been several outbreaks since 1974, which have all been linked to heavy rainfall, followed by increased mosquito activity [19,20]. WNV_KUN_ is endemic in Northern and Central Australia, but it was considered to be of limited medical and veterinary importance until 2011, when it caused an equine encephalitis outbreak in south-eastern Australia. During that outbreak, the fatality rate was as high as 15–20% amongst clinically affected horses, including horses terminated for humane reasons [17,21]. Similar to MVEV, clinical cases of WNV infection have occurred in humans and horses following heavy rainfall, causing flooding and leading to favourable conditions for mosquito activity. Recently, WNV_KUN_ was detected by RT-PCR in samples from saltwater crocodiles, in which the virus causes skin lesions that lead to hide depreciation [3,22,23]. While WNV_KUN_ does not cause any apparent systemic diseases in saltwater crocodiles, there remains the potential for more virulent WNV strains to emerge, as has happened in the Americas during the past two decades [24,25].

KOKV infections have been reported in humans in Australia [26]. The clinical presentation for KOKV infection is similar to DENV fever; therefore, clinical diagnosis should be accompanied by laboratory testing [27]. In addition to fever and headache, infected individuals may experience influenza-like aches, myalgia, polyarthralgia syndrome, and polyarthritis [15,16,26,27].

Since its isolation in 1961 at Edge Hill, a suburb of Cairns in Northern Queensland, there has been only one report of EHV infection in humans, in which the clinical picture was characterised by myalgia, arthralgia, and fatigue [28]. Anti-EHV antibodies have been detected in wallabies, kangaroos, and bandicoots; thus, EHV is generally thought to be a virus of marsupials [29].

Antibodies to ALFV, FRV, SEPV, and STRATV have also been detected in patients who tested positive for other flaviviruses [16,26,30]. Further investigation is needed to confirm whether the neutralising antibody titres were due to actual infection or cross-reactivity with other flaviviruses. Currently, there is no evidence of human exposure to NMV; thus, it likely poses a low risk to humans [31]. Generally, individuals infected with most flaviviruses have low levels of viraemia, sometimes below detectable levels, even in clinically ill patients [32]. Therefore, serology remains the sole diagnostic method in such cases, with the virus neutralisation test being the gold standard diagnostic method [22,33].

Following our previous study demonstrating the role of WNV_KUN_ in saltwater crocodile skin lesions [3], this investigation aimed to identify other flaviviruses that might infect saltwater crocodiles in northern Australia. Furthermore, an understanding of the potential role of crocodiles in the transmission cycle of flaviviruses and possible serological cross-reactivity between various flaviviruses, which might interfere with the specificity of flavivirus diagnostics in crocodiles and response to a WNV vaccine, is of public health and industry interest.

## 2. Materials and Methods

### 2.1. Study Area

The samples used in this study were collected from three commercial saltwater crocodile farms (Farms A, B, and C), all located in the greater Darwin region of the Northern Territory, Australia.

### 2.2. Animals, Blood Sample Collection, and Processing

Blood samples were collected from 500, predominantly male, farmed saltwater crocodiles at harvest when the animals were 3–4 years old. None of the sampled crocodiles had a history of clinical diseases. During harvest, crocodiles were captured using an electrical stunner that temporarily rendered the crocodile immobile and unconscious, in order to allow for safe handling, as previously described [34,35]. After immobilisation, blood was aseptically collected from the occipital sinus using 18” needles and plain (without anticoagulant) vacutainer tubes (Becton Dickinson, Franklin Lakes, NJ, USA). Blood was allowed to clot at room temperature, and it was centrifuged at room temperature for 10 min at 1000 rpm. Collected serum samples were aliquoted in triplicate into sterile Eppendorf tubes and immediately stored at −20 °C. Serum samples were heat-inactivated at 56 °C for 30 min before they were tested via blocking ELISA and virus neutralisation.

### 2.3. Anti-Flavivirus Blocking ELISA

The 500 crocodile serum samples were screened for the presence of flavivirus antibodies by a competitive blocking ELISA, as previously described [33,36]. Wells of Serocluster^TM^ U-bottom high-binding 96-well plates (Sigma Aldrich Pty Ltd. St. Louis, MO, USA) were coated with a pre-determined dilution of WNV_KUN_ antigen (infected C6/36 cell lysate) in carbonate coating buffer (50 µL per well) and incubated overnight at 4 °C. The plates were then washed four times with phosphate-buffered saline (PBS) with 0.05% Tween-20, and nonspecific binding sites were blocked with blocking buffer (0.05 M Tris, 1 mM EDTA, 0.15 M NaCl, 0.05% (*v*/*v*) Tween-20, 0.2% (*w*/*v*) casein, and pH 8.0), followed by one-hour incubation at room temperature. On a separate plate, 5-fold dilution in the blocking buffer was performed for all the samples, including the positive and negative controls. Fifty µL of the diluted samples were transferred to the pre-coated plates in duplicate and incubated for one hour at 28 °C. Then, 50 µL of the anti-pan-flavivirus envelope protein-specific monoclonal antibody (mAb), 6B6C-1 [37], was added to the plates on top of the serum samples. The plates were incubated for a further 1 h at 28 °C. Following four washes, as above, a horseradish peroxidase (HRP)-conjugated goat anti-mouse polyclonal antibody, pre-adsorbed against human immunoglobulins and fetal bovine serum (Jackson ImmunoResearch Laboratories, West Grove, PA, USA) and diluted in the blocking buffer, was added to the plates. Following a one-hour incubation at 28°C, the plates were washed six times. The plates were then thoroughly washed, and the antibody conjugate binding was visualised via the addition of 100 µL substrate buffer (citrate-phosphate buffer, pH 4.2 supplemented with 1 mM (2,2′-azino-bis (3-ethylbenzthiazolinesulfonic acid); ABTS), and 3 mM H_2_O_2_) and incubating the plates in the dark for one hour. The enzyme activity was quantified by measuring the optical density (OD) at a wavelength of 405 nm in an automated ELISA plate reader (SpectraMax^®^ 190 Microplate Reader, Molecular Devices, LLC, San Jose, CA, USA). The percentage of the inhibition of binding of the mAb 6B6C-1 was calculated using the following formula [36]:

Percentage of inhibition = 100 − (TS-B)/(CS-B)] × 100

where TS = OD of test serum, CS = OD of control serum, and B = background OD. Samples with an inhibition of ≥30% were considered positive. In some instances, samples giving borderline results were retested for confirmation.

### 2.4. Virus Culture

Nine viruses were screened in this study. These included MVEV (strain 1–51), KOKV (strain MRM32), STRATV (strain C338), ALFV (strain CY2269), FRV (strain K78296), WNV-KUNV (NSW2011), NMV (CY1014), EHV (C281), and SEPV (MK7979) (Table 1). All virus stocks were titrated by the TCID_50_ method, as previously described [38]. Briefly, each virus stock was diluted 10-fold in Dulbecco’s modified Eagle’s medium (DMEM; Thermofisher Scientific, Seventeen Mile Rocks, Qld, Australia) supplemented with 2% fetal bovine serum (FBS) (Gibco, Sigma Aldrich Pty Ltd., St. Louis, MO, USA), one-time penicillin–streptomycin, and 2 mM L-glutamine (Gibco, Gibco, Sigma Aldrich Pty Ltd., St. Louis, MO, USA). The diluted virus was transferred into a 96-well tissue culture plate (Costar, Corning), pre-seeded with baby hamster kidney cells (BSR) in DMEM supplemented with 5% FBS, one-time penicillin (50 U/mL), streptomycin (50 µg/mL), and 2 mM L-glutamine (Gibco, Sigma Aldrich Pty Ltd., St. Louis, MO, USA). The plates were incubated at 37 °C and 5% CO_2_ for five days, after which the plates were scored for cytopathic effect (CPE). The cell monolayers were fixed with 20% acetone supplemented with 0.02% (*w*/*v*) bovine serum albumin (BSA) by overnight incubation at 4 °C. The fixative was then discarded, and the plates were dried overnight at room temperature. ELISA was performed on fixed plates using the mAb 4G2 (anti-flavivirus E protein), as previously described [3,33,39]. The virus titres were expressed in TCID_50_/mL, as previously described [38,40].

**Table 1:** Summary of viruses used.

Virus.	Strain	Accession Number	Location of Isolation	Source of Isolate	Year of Isolation	Propagation Cell Line	Reference
MVEV	MVE-1-51	AF161266.1	Mooroopna, Victoria	Fatal human case	1951	C6/36	[41]
KOKV	MRM32 strain	L48969.1	Kowanyama, QLD	*C. annulirostris* and *C. pullus*	1960	C6/36	[42]
STRATV	STRV C338	KM225263.1	Cairns, QLD	*Aedes vigilas*	1961	C6/36	[43]
ALFV	CY2269	-	Pompuraaw, QLD	*Culex annulirostris*	1999	C6/36 and PS-EK	[44,45]
FRV	K78296	-	Fitzroy Crossing, QLD	*Ochlerotatus normanensis*	2011	C6/36	[30]
WNV	NSW2011	JN887352.1	New South Wales	Horse brain	2011	C6/36	[46]
NMV	CY1014	KC788512.1	New Mapoon, QLD	*Culex annulirostris*	1998	PS-EK	[47]
EHV	C281	AF275876.2	Edge Hill (Cairns) QLD	*Culex annulirostris*	1961	C6/36	[48]
SEPV	MK7979	NC_008719.1	Sepik, PNG		1966	PS-EK	[49]

### 2.5. Virus Neutralisation Assays

Crocodile serum samples that were positive in the 6B6C-1 mAb blocking ELISA were tested in virus micro-neutralisation assays using the baby hamster kidney-derived cell line (BSR). Serum samples were titrated in doubling dilution down the plate (1:20 to 1:2560), which was made in plain DMEM. The virus was diluted in DMEM supplemented with 2% FBS, one-time penicillin–streptomycin, and two mM L-glutamine, with a concentration of 100 TCID_50_ units in 50 µL. Fifty µL of the suspension was added to each well in the 96-well plates, and the plates were incubated at 37 °C and 5% CO_2_ for one hour. After a one-hour incubation, 1 × 10^4^ BSR cells, resuspended in 50 µL in the above-mentioned cell culture medium, were added to each well of the 96-well plates. The plates were incubated at 37 °C in a humidified CO_2_ incubator for five days. Then, the plates were scored for CPE, and the cells were fixed with the 20% acetone fixative buffer, as described above. Fixed-cell ELISA was performed using mAb 4G2 to identify virus-infected wells, and the neutralising titre was expressed as the reciprocal of the highest serum dilution where the virus replication was neutralised, i.e., there was no virus replication [36,39].

### 2.6. Data Analysis

Data analysis and generation of graphs were performed using GraphPad Prism 9 (GraphPad Software, Inc., San Diego, CA, USA) for Windows (version 9.2.0 (332) 2020). Neutralising antibody titres were presented as a reciprocal value corresponding to 20 being the lowest dilution and 2560 being the highest.

The criterion of four-fold greater neutralising antibody titre was used to take the potential cross-reactivities that may lead to false-positive results, in the case of samples positive for two or more flaviviruses, into account [50].

## 3. Results

### 3.1. Sample Set Description

A total of 500 serum samples were collected, of which 150 samples were collected from Farm A, 200 from Farm B, and 150 from Farm C, all of which were located near Darwin in the Northern Territory, Australia (Table S1).

### 3.2. Blocking ELISA Results

**Figure 1:**
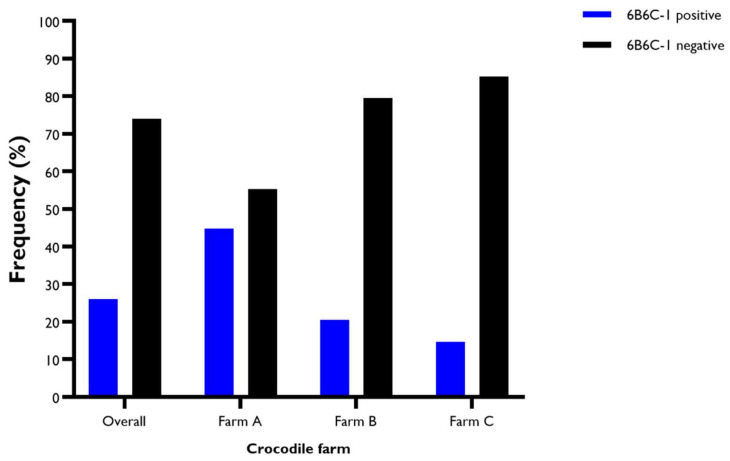
Sero-prevalence of flavivirus antibodies in farmed crocodiles based on blocking ELISA using the flavivirus envelope protein-specific mAb, 6B6C-1. Positive samples are blue bars, and negative samples are black bars.

By pan-flavivirus blocking ELISA with the 6B6C-1 mAb, 130 (26%, *n* = 500) samples tested positive. From the 130 positive samples, 67 (44.7%, *n* = 150) were from Farm A, with 20.5% (*n* = 200) from Farm B and 14.7% (*n* = 150) from Farm C (Figure 1 and Table S1).

### 3.3. Virus Neutralisation Assay

When the subset (*n* = 130) of the samples that were positive in the 6B6C-1 blocking ELISA were tested using VNT, 76 samples (58.5%) had neutralising antibodies to FRV. Of these, 35 (52.2%, *n* = 67) samples were from Farm A, 25 (60.9%, *n* = 41) were from Farm B, and 16 (72.7%, *n* = 22) were from Farm C. Fifty-three of the FRV positive samples had a neutralising titre of 20, 21 samples had a titre of 40, three samples had a titre of 80, and three samples had a titre of 160. Forty-one samples (31.5%, *n* = 130) had neutralising activity for WNV. Of the 41 samples, 29 (43.3%, *n* = 67) were from Farm A, 9 (22.0%, *n* = 41) from Farm B, and 3 (13.6%, *n* = 22) from Farm C. The WNV neutralising titres varied between 20 and 2560. Twenty-four (18.5%, *n* = 130) samples had neutralising antibodies to STRATV, of which 22 (16.9%, *n* = 67) came from Farm A, with one from Farm B (4.5%, *n* = 41) and one from Farm C (4.5%, *n* = 22). SEPV was the fourth most frequent of the tested flaviviruses at nine (6.9%, *n* = 130). At the farm level, neutralising antibodies to SEPV were detected in six samples (9.0%, *n* = 67) at Farm A, with two samples (4.9%, *n* = 41) at Farm B and one sample (4.5%, *n* = 22) at Farm C. The highest titre observed was 640, in a sample from Farm A. Neutralising antibodies to MVEV, EHV, and ALFV were each present in two samples (1.5%, *n*=130), and they were all from Farm A. Neutralising antibodies to KOKV and NMV were each present in one sample (0.8%, *n* = 130), both from Farm A.

**Figure 2:**
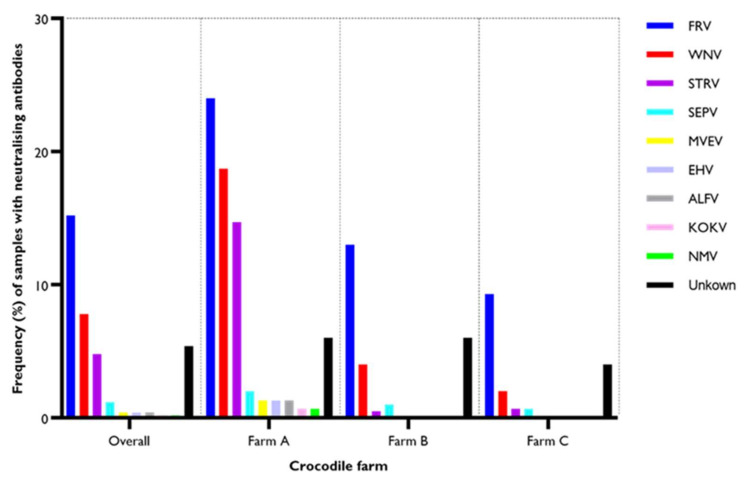
Proportion of samples that have neutralising antibodies against various viruses tested in the virus neutralisation assay. The overall frequency was determined based on the 500 samples collected from three crocodile farms. The frequency per farm was estimated based on *n* = 150 at Farm A, *n* = 200 at Farm B, and *n* = 150 at Farm C. Of the 130 samples positive in the 6B6C-1 blocking ELISA, 27 (20.8%, *n* = 130) did not have neutralising antibodies to any of the tested viruses. They were designated as unknown flaviviruses. Farm B had the highest number of animals positive for unknown flaviviruses (*n* = 12), followed by Farm A (*n* = 9) and Farm C (*n* = 6) (Figure 3 and Table S1).

Overall, based on the initial sample size of 500, composed of 150 samples from Farm A, 200 from Farm B, and 150 from Farm C, samples with neutralising antibodies to FRV were the most frequent, followed by WNV. The least frequently detected viruses were KOKV and NMV (Figure 2).

**Figure 4:**
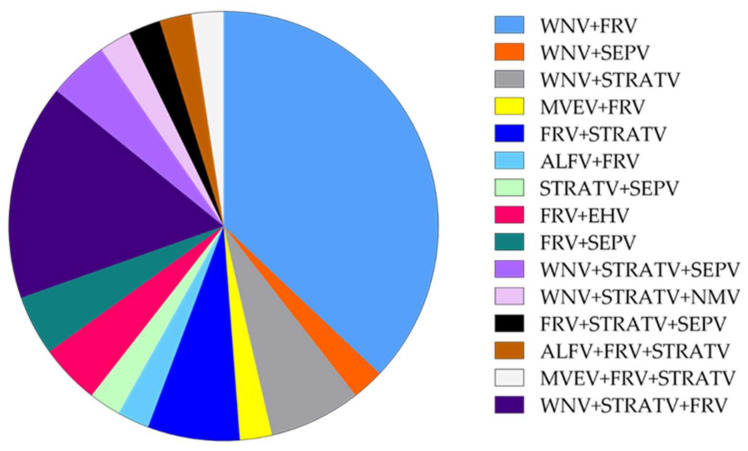
Proportion (%) of samples positive in the 6B6C-1 blocking ELISA that have neutralising antibodies against two or more tested flaviviruses. The overall frequency was determined based on *n* = 130. The frequency per farm was estimated based on *n* = 67 at Farm A, *n* = 41 at Farm B, and *n* = 22 at Farm C (see Table S2 in Supplementary Materials).

Thirty-nine samples had neutralising antibodies to more than one of the flaviviruses tested for (Figure 4 and Table S2). Twenty-six samples (20%, *n* = 130) had neutralising antibodies to any two tested flaviviruses, while 13 (10%, *n* = 130) had neutralising antibodies to any three of the tested flaviviruses.

The verdict (diagnosis) for VNT was based on the analysis of the set of neutralising antibodies to the virus from the same serogroup. Out of the 130 flavivirus-positive samples, 16 samples (12.3%) had neutralising antibodies to both WNV and FRV, three samples (2.3%) had antibodies to WNV and STRATV, three samples (2.3%) had antibodies to FRV and STRATV, two samples (1.5%) had antibodies to FRV and EHV, one sample (0.8%) had antibodies to WNV and SEPV, one sample (0.8%) had antibodies to ALV and FRV, one sample (0.8%) had antibodies to STRATV and SEPV, one sample (0.8%) had antibodies to FRV and SEPV, and one sample (0.8%) had antibodies to MVEV and FRV.

Seven samples out of the 130 flavivirus-positive sera (5.4%) had antibodies to FRV, WNV, and STRATV, two samples (1.5%) had antibodies to WNV, STRATV, and SEPV, one sample (0.8%) had antibodies to NMV, STRATV, and WNV, one sample (0.8%) had antibodies to FRV, STRATV, and SEPV, one sample (0.8%) had antibodies to ALV, FRV, and STRATV, and one sample (0.8%) had antibodies to FRV, MVEV, and STRATV.

Cross-reactivity between viruses of the same serogroup was often observed, as opposed to coinfection (Table S1). For example, sample A131 was positive for ALFV, with a neutralisation titre of 320 and comparably low titres of 20 (16-fold less) to the closely related flaviviruses MVEV and WNV. This trend was observed for other flaviviruses of the same serogroup (Table S1). In contrast, we observed cases of possible coinfection, where samples had similar neutralising antibody titres to viruses of the same serogroup or distant serogroups. Sample A69 gave, by far, the strongest neutralising antibody titre to SEPV at 640, but it also had strong neutralising titres against WNV, FRV, and STRATV. There were more examples of such instances (Table S1).

## 4. Discussion

WNV has been reported in crocodilians, including alligators (*Alligator mississippiensis*) in the Americas, Nile crocodiles (*Crocodylus niloticus*) in Africa, Israel, and Mexico [51,52], and saltwater crocodiles (*Crocodylus porosus*) in Australia [3,23]. This is the first study to provide serological evidence of infections with zoonotic flaviviruses, other than WNV, in saltwater crocodiles in Australia. Apart from WNV, none of the other flaviviruses tested for have ever been reported in crocodilian species.

Most serological studies, so far, have focused on mammalian and avian species; thus, this is the first study of the seroepidemiology of flaviviruses infection in reptilians, and it included a substantial sample size. Antibodies to most of the flaviviruses tested in this study have been detected in other hosts, mostly avian and mammalian species. However, while data from this study provides general information on zoonotic flaviviruses circulating in farmed saltwater crocodiles in the Northern Territory, it does not necessarily represent the true prevalence of these viruses. Considering that flavivirus infections are seasonally- and locality-dependent, understanding the true prevalence, dynamism, and risk factors of the tested viruses would require sampling crocodiles of different ages and sex in different seasons, with varying rainfall and more locations, as well as sampling wild-caught crocodiles. Since crocodiles are farmed for skin and hides and harvested when they are three to four years old, they would have been through at least three wet seasons.

Most of the studied flaviviruses have been associated with clinical and subclinical infections in various hosts. These hosts include humans, birds, horses, and European rabbits [53]. Antibodies to ALFV, FRV, and STRATV have been detected in humans, horses, and cattle [30,54]. In contrast, the detection of neutralizing antibodies for NMV is puzzling, given that no vertebrate host has been identified since its isolation from the *C. annulirostris* mosquitoes collected in 1998 on the Cape York Peninsula, Queensland [55]. It would be important to investigate the main host(s) for this virus.

Intriguingly, some samples had neutralising antibodies to SEPV, a virus that has never been reported in Australia. The virus was first isolated from the *Mansonia septempunctata* collected from the Sepik district of Papua New Guinea (PNG). The virus was then isolated in 1998 from Balimo in Western Province of PNG, a place relatively closer to Australian mainland [56]. Since then, the virus has been thought to be restricted to PNG [57]. While the spread of flaviviruses from one location to another can result from international travel, there would always need to be a vector of the virus that would sustain the infection locally. *Mansonia septempunctata*, the mosquito vector for SEPV, is not known to occur in the Northern Territory [58]. Most SEPV-positive samples had a neutralising antibody titre varying between 20 and 40. Additionally, three-quarters of the samples positive for SEPV, which had a neutralising titre of 40 and above, were positive for FRV, another flavivirus belonging to the YFV serogroup. Moreover, another virus of the YFV serogroup, the Bamaga virus (BgV), has been reported in Australia [59]. While this virus was not tested for in this study, the possibility of it infecting crocodiles cannot be ruled out, since infection in vertebrates was reported earlier [59,60]. Whether the detection of neutralising antibodies to SEPV is linked to an infection or result of cross-reactivity with another flavivirus of the YFV serogroup, such as FRV, BgV, or any other unknown flavivirus, would require further investigation.

It is not unexpected that a relatively high number of samples tested positive for two or more flaviviruses. This scenario can be explained by the fact that most of the studied flaviviruses are transmitted by the same vectors [61]. Both MVEV and WNV, the most clinically significant flaviviruses in Australia, are mainly transmitted by *Culex annulirostris*. Similarly, KOKV and NMV have also been isolated from *Cx. annulirostris*, which is abundant in the Northern Territory and, therefore, likely to have transmitted these viruses to the crocodiles. STRV has been isolated from, and is transmitted by, *Aedes* spp. (*Aedes aculeatus*, *Ae. alternans*, *Ae. notoscriptus*, *Ae. procax*, and *Ae. vigilax*) and *Anopheles annulipes* [61,62,63]. EHV has also been isolated from two mosquito species, including *Cx. annulirostris* and *Ae. vigilax* [64]. Neutralising antibodies to FRV were the most frequently detected in analysed samples. This finding is not surprising, considering the ecology of FRV and its principal vector *Ae. normanensis*, a mosquito species that is abundantly prevalent in northern Australia. The maintenance of the *Ae. normanensis* population requires dry areas, and its activity can significantly increase after rainfall. Thus, these environmental conditions are fulfilled in northern Australia [30,65].

Another possibility for the occurrence of antibodies to a variety of flaviviruses is the cross-reactive immunity among flaviviruses from the same serogroup [66]. Some samples that were positive for WNV were also positive for MVEV and ALFV. The same phenomenon was observed for flaviviruses from the KOKV and YFV groups (Table S1). Since the criteria of a four-fold greater neutralising antibody titre was used to take the potential cross-reactivities into account [50], co-infection and superinfection with various flaviviruses are likely possible in saltwater crocodiles.

The high occurrence of samples with neutralising antibodies to FRV and occurrence of those with dual neutralising antibodies to FRV and WNV are interesting findings that should be investigated further, in the context of the development of diagnostic tools for WNV [67]. The relatively high occurrence of samples positive in the pan-flavivirus ELISA using mAb 6B6C-1, but with no neutralising antibodies to any of the tested flaviviruses, suggests the circulation of unknown vertebrate infecting flaviviruses, which also infect saltwater crocodiles. This finding could be further explored via elucidation of the virome of the crocodiles and mosquitoes abundantly found on these farms, using deep sequencing and transcriptomic analysis [68].

The main limitations of this study were the similar age of all the animals and predominance of males. Crocodiles are harvested for their hides when they reach a particular size, primarily between three to four years of age. Due to the lack of sex chromosomes, crocodile sex is determined by egg incubation temperature, which happen to favour males. Additionally, most crocodile farmers prefer rearing faster-growing males, which was probably the case with the cohort we sampled in this study [69,70]; therefore, it was impossible to investigate the additional risk factors associated with the seroconversion to the studied flaviviruses. Hence, while this study gives a snapshot of the flaviviruses circulating in crocodiles in the Northern Territory of Australia, it is not necessarily a complete representation of the prevalence of the studied mosquito-borne flaviviruses. To better investigate the dynamics, it would be necessary to also study ecological factors, such as season, rainfall (including draught events), and humidity [71], as well as wild versus farmed crocodiles. A longitudinal study sampling crocodiles hatched and or slaughtered in different seasons and additional regions, such as northern Queensland, might shed further light on the various risk factors.

## 5. Conclusions

The present study shows that farmed crocodiles from the Northern Territory are exposed to a variety of zoonotic flaviviruses, other than WNV. The detection of neutralising antibodies to more than one virus in one host suggests co-infection with different viruses at a given time point or animals that were consecutively exposed during their lifetime. Considering the level of neutralising titres for some viruses, it would be worth investigating the pathogenesis of infection in crocodiles with the viruses of “One Health” importance, notably MVEV. Studying the pathogenesis will help our understanding of the role of crocodiles in the transmission cycle, i.e., whether they can develop a viremia high enough to sustain the transmission cycle or are dead-end hosts.

Most of the viruses used in this study have been linked to clinical and subclinical infections in humans in Australia and other Pacific regions; thus, they all are clinically significant in the ”One Health” context. Therefore, saltwater crocodile can be used as a sentinel species to monitor arboviral infection dynamics and predict flaviviruses outbreaks in humans and animals using the “One Health” approach.

## Figures and Tables

**Figure 3:**
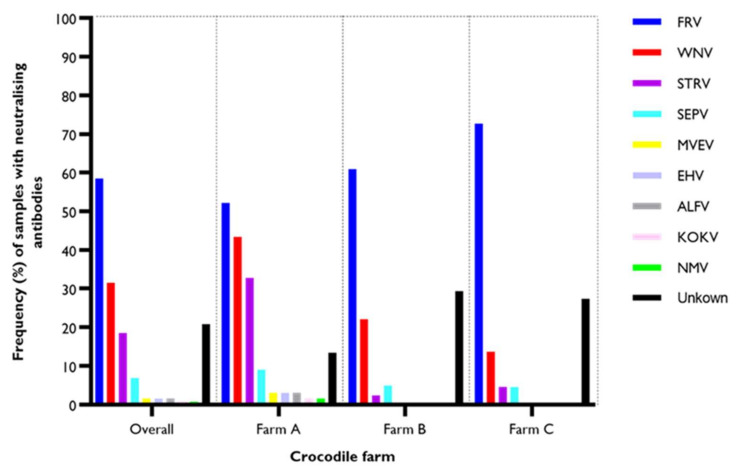
Proportion of samples positive in the 6B6C-1 blocking ELISA that have neutralising antibodies against tested flaviviruses. The overall frequency was determined based on *n* = 130. The frequency per farm was estimated based on *n* = 67 at Farm A, *n* = 41 at Farm B, and *n* = 22 at Farm C.

## Data Availability

All relevant data are included in the report and supplementary data.

## Supplementary Materials

The following supporting information can be downloaded at: https://www.mdpi.com/article/10.3390/v14051106/s1. Table S1: Summary data of 6B6C-1 blocking ELISA and virus neutralisation test. Table S2: Frequency of co-infection or cross-reactivity among tested samples.
