# Supplementary material for: Evidence of Infection with Zoonotic Mosquito-Borne Flaviviruses in Saltwater Crocodiles (Crocodylus porosus) in Northern Australia
Source: Viruses. 2022 May 21;14(5):1106. doi: 10.3390/v14051106 (PMC9144604; doi:10.3390/v14051106)
Supplement: Supplementary file 1 [file viruses-14-01106-s001.zip › Table S1. Summary data of 6B6C-1 blocking ELISA and virus neutralisation test 26-04-22.pdf]

Summary data of 6B6C-1 blocking ELISA and virus neutralisation test

SN	Samples	Farm	Percentage of inhibition	Virus neutralising antibody titres									VNT Results
			6B6C-1	WNV	MVEV	ALFV	FRV	SEPV	EHV	KOKV	STRATV	NMV	
1	A02	Farm A	86.41	320	20	0	40	20	0	0	0	0	WNV, FRV
2	A03	Farm A	38.85	160	0	0	0	20	0	0	0	0	WNV, SEPV
3	A04	Farm A	60.00	40	0	0	20	0	0	0	0	0	WNV, FRV
4	A06	Farm A	82.20	640	20	0	20	0	0	0	80	0	WNV, STRATV
5	A07	Farm A	70.15	ND	0	0	20	0	0	0	80	0	FRV, STRATV
6	A09	Farm A	74.25	0	0	0	40	20	0	0	80	0	FRV, STRATV
7	A23	Farm A	84.40	40	0	0	0	0	0	0	0	0	WNV
8	A27	Farm A	60.81	0	0	0	0	0	0	0	0	0	unknown
9	A28	Farm A	35.57	0	0	0	20	0	0	0	0	0	FRV
10	A29	Farm A	50.07	0	0	0	20	0	0	0	0	0	FRV
11	A30	Farm A	36.93	0	0	0	20	0	0	0	0	0	FRV
12	A31	Farm A	44.68	0	0	0	0	0	0	0	0	0	unknown
13	A32	Farm A	33.62	0	0	0	20	0	0	0	0	0	FRV
14	A33	Farm A	37.14	0	0	0	20	0	ND	0	20	0	FRV, STRATV
15	A35	Farm A	44.77	20	0	0	20	0	0	0	0	0	WNV, FRV
16	A36	Farm A	69.80	0	0	0	20	0	0	0	0	0	FRV
17	A37	Farm A	73.18	0	0	40	20	0	0	0	0	0	ALFV, FRV
18	A41	Farm A	41.15	0	0	0	0	0	0	0	0	0	unknown
19	A42	Farm A	62.41	0	ND	0	20	0	0	0	0	0	FRV
20	A47	Farm A	31.65	0	0	0	0	0	0	20	0	0	KOKV
21	A52	Farm A	68.10	40	ND	0	20	ND	0	0	0	0	WNV, FRV
22	A53	Farm A	32.50	20	0	0	0	0	0	0	0	0	WNV
23	A54	Farm A	52.90	80	0	0	160	0	0	0	80	0	WNV, FRV, STRATV
24	A56	Farm A	67.04	160	0	0	160	ND	0	0	0	0	WNV, FRV
25	A57	Farm A	35.32	0	0	0	0	0	0	0	20	0	STRATV
26	A58	Farm A	64.33	0	0	0	20	0	0	0	0	0	FRV
27	A59	Farm A	50.47	0	ND	0	40	0	0	0	0	0	FRV
28	A63	Farm A	51.86	0	0	0	0	ND	0	0	0	0	unknown
29	A65	Farm A	41.10	0	0	0	0	ND	0	0	0	0	unknown
30	A66	Farm A	38.32	80	0	0	40	ND	0	0	80	0	WNV, STRATV, FRV
31	A68	Farm A	43.18	40	0	0	0	0	0	0	0	0	WNV
32	A69	Farm A	50.16	320	ND	0	160	640	0	0	160	0	WNV, STRATV, SEPV
33	A71	Farm A	37.72	0	0	0	0	20	20	0	40	0	STRATV, SEPV
34	A75	Farm A	55.22	0	0	0	0	20	0	0	0	0	SEPV
35	A76	Farm A	49.80	0	0	0	0	0	0	0	0	0	unknown
36	A77	Farm A	39.04	20	0	0	20	0	0	0	80	0	WNV, FRV, STRATV
37	A78	Farm A	51.79	0	40	0	40	ND	0	0	0	0	MVEV, FRV
38	A79	Farm A	73.87	80	0	0	40	0	0	0	80	0	WNV, FRV, STRATV
39	A80	Farm A	78.34	80	0	0	40	0	0	0	0	0	WNV, FRV
40	A82	Farm A	57.79	20	0	0	0	0	0	0	0	0	WNV
41	A83	Farm A	34.90	20	0	0	20	0	0	0	80	0	WNV, FRV, STRATV
42	A85	Farm A	56.82	0	0	0	40	0	0	0	0	0	FRV
43	A86	Farm A	72.76	1280	0	0	20	40	ND	0	40	0	WNV, STRATV, SEPV
44	A92	Farm A	89.29	80	0	0	0	0	0	0	40	0	WNV, STRATV
45	A93	Farm A	63.03	0	0	0	20	ND	0	0	0	0	FRV

46	A95	Farm A	75.56	40	0	0	40	20	0	0	0	0	WNV, FRV
47	A97	Farm A	32.64	20	0	0	0	0	0	0	0	0	WNV
48	A98	Farm A	38.40	0	0	0	20	0	20	0	0	0	FRV, EHV
49	A99	Farm A	61.52	0	0	0	0	0	ND	0	0	0	unknown
50	A100	Farm A	55.25	0	0	0	20	0	20	0	ND	0	FRV, EHV
51	A104	Farm A	76.02	2560	0	0	0	ND	0	0	0	0	WNV
52	A108	Farm A	38.79	80	20	0	20	0	ND	0	40	0	WNV, FRV, STRATV
53	A114	Farm A	61.05	0	0	0	20	0	0	0	0	0	FRV
54	A115	Farm A	77.91	2560	20	0	20	0	ND	0	160	80	WNV, STRATV, NMV
55	A116	Farm A	48.82	2560	0	0	80	ND	0	0	0	0	WNV
56	A122	Farm A	47.21	0	ND	0	20	20	0	0	0	0	FRV, SEPV
57	A123	Farm A	34.66	0	0	0	0	0	0	0	0	0	unknown
58	A127	Farm A	74.99	20	ND	0	20	ND	0	0	20	0	WNV, FRV, STRATV
59	A129	Farm A	43.15	0	0	0	0	0	0	0	20	0	STRATV
60	A131	Farm A	68.00	20	20	320	20	ND	0	0	80	0	ALFV, FRV, STRATV
61	A135	Farm A	48.03	20	0	0	0	0	0	0	0	0	WNV
62	A137	Farm A	56.71	20	80	0	40	20	0	0	80	0	MVEV, FRV, STRATV
63	A139	Farm A	40.94	0	0	0	0	0	0	0	40	0	STRATV
64	A146	Farm A	49.85	20	0	0	0	ND	0	0	0	0	WNV
65	A148	Farm A	54.98	20	0	0	0	0	0	0	80	0	WNV, STRATV
66	A149	Farm A	30.32	0	ND	0	0	0	0	0	0	0	unknown
67	A150	Farm A	51.32	0	ND	0	20	0	0	0	0	0	FRV
68	A157	Farm B	41.91	20	0	0	0	0	0	0	0	0	WNV
69	A158	Farm B	63.91	40	0	0	20	0	0	0	0	0	WNV, FRV
70	A159	Farm B	51.34	40	0	0	0	0	0	0	0	0	WNV
71	A160	Farm B	46.49	80	0	0	0	0	0	0	0	0	WNV
72	A161	Farm B	47.25	0	0	0	0	0	0	0	0	0	unknown
73	A163	Farm B	43.21	0	0	0	0	0	0	0	0	0	unknown
74	A164	Farm B	49.44	0	0	0	0	0	0	0	0	0	unknown
75	A165	Farm B	65.29	0	0	0	0	0	0	0	0	0	unknown
76	A166	Farm B	42.37	0	0	0	0	0	0	0	0	0	unknown
77	A167	Farm B	43.99	40	0	0	20	0	0	0	0	0	WNV, FRV
78	A170	Farm B	51.60	0	0	0	20	0	0	0	0	0	FRV
79	A171	Farm B	49.20	0	0	0	20	0	0	0	0	0	FRV
80	A182	Farm B	50.04	0	0	0	20	0	0	0	0	0	FRV
81	A206	Farm B	45.33	20	0	0	20	0	0	0	0	0	WNV, FRV
82	A209	Farm B	50.08	0	ND	0	0	0	0	0	0	0	unknown
83	A217	Farm B	37.50	0	0	0	20	0	0	0	0	0	FRV
84	A229	Farm B	41.26	0	0	0	40	0	0	0	0	0	FRV
85	A231	Farm B	45.24	0	0	0	0	0	0	0	0	0	unknown
86	A235	Farm B	50.80	0	0	0	0	0	0	0	0	0	unknown
87	A236	Farm B	56.89	40	0	0	20	ND	0	0	0	0	WNV, FRV
88	A237	Farm B	56.32	0	0	0	20	0	0	0	0	0	FRV
89	A238	Farm B	53.56	0	0	0	20	0	0	0	0	0	FRV
90	A239	Farm B	58.07	0	0	0	0	0	0	0	0	0	unknown
91	A240	Farm B	46.20	0	0	0	0	0	0	0	0	0	unknown
92	A243	Farm B	54.06	0	0	0	20	20	0	0	0	0	FRV, SEPV
93	A244	Farm B	34.84	0	0	0	40	0	0	0	0	0	FRV
94	A245	Farm B	45.55	0	0	0	80	0	0	0	0	0	FRV
95	A246	Farm B	41.65	0	0	0	40	20	0	0	0	0	FRV

96	A247	Farm B	39.92	0	0	0	80	0	0	0	0	0	FRV
97	A248	Farm B	47.13	0	0	0	40	0	ND	0	0	0	FRV
98	A249	Farm B	35.12	0	0	0	40	20	0	0	0	0	FRV
99	A254	Farm B	32.70	0	0	0	40	0	0	0	0	0	FRV
100	A266	Farm B	72.31	40	0	0	40	20	0	0	0	0	WNV, FRV
101	A280	Farm B	43.06	0	0	0	40	0	0	0	0	0	FRV
102	A281	Farm B	32.40	0	0	0	0	0	0	0	0	0	unknown
103	A282	Farm B	71.92	0	0	0	20	0	0	0	0	0	FRV
104	A286	Farm B	47.31	0	0	0	0	0	ND	0	0	0	unknown
105	A293	Farm B	36.98	0	ND	0	40	0	0	0	0	0	FRV
106	A337	Farm B	45.36	80	20	0	40	0	0	0	0	0	WNV, FRV
107	A342	Farm B	41.88	0	0	0	20	0	0	0	0	0	FRV
108	A343	Farm B	45.46	0	0	0	40	40	0	0	20	0	FRV, STRATV, SEPV
109	A391	Farm C	55.07	0	0	0	0	0	0	0	0	0	unknown
110	A392	Farm C	53.97	0	0	0	20	0	0	0	0	0	FRV
111	A393	Farm C	52.11	0	0	0	20	0	0	0	0	0	FRV
112	A394	Farm C	53.86	0	0	0	20	0	0	0	0	0	FRV
113	A395	Farm C	56.03	40	0	0	20	0	0	0	0	0	WNV, FRV
114	A396	Farm C	60.78	0	0	0	20	0	0	0	0	0	FRV
115	A397	Farm C	52.95	0	0	0	20	0	0	0	0	0	FRV
116	A398	Farm C	51.83	0	0	0	20	0	0	0	0	0	FRV
117	A399	Farm C	54.67	0	0	0	20	0	0	0	0	0	FRV
118	A400	Farm C	55.38	0	0	0	0	0	0	0	0	0	unknown
119	A401	Farm C	54.47	0	0	0	0	0	0	0	0	0	unknown
120	A402	Farm C	46.12	20	0	0	20	0	0	0	0	0	WNV, FRV
121	A403	Farm C	48.54	0	0	0	20	0	0	0	0	0	FRV
122	A404	Farm C	42.72	0	0	0	20	0	0	0	0	0	FRV
123	A405	Farm C	46.84	0	0	0	20	0	0	0	0	0	FRV
124	A406	Farm C	54.62	0	0	0	0	0	0	0	0	0	unknown
125	A407	Farm C	53.56	20	0	0	20	0	0	0	0	0	WNV, FRV
126	A408	Farm C	52.73	0	0	0	0	0	0	0	0	0	unknown
127	A409	Farm C	54.73	0	0	0	0	0	0	0	0	0	unknown
128	A410	Farm C	52.88	0	0	0	20	0	0	0	0	0	FRV
129	A440	Farm C	47.35	0	ND	0	0	40	0	0	0	0	SEPV
130	A473	Farm C	42.27	0	0	0	0	0	0	0	160	0	STRATV

ND (not determined) virus neutralisation was inconsistent at the lowest serum dilution and a titre could not be determined

Unknown these samples were positive in panflaviviruses blocking ELISA (6B6C-1) but did not have neutralising antibodies to any of the tested viruses
