# Supplementary material for: Evidence of Infection with Zoonotic Mosquito-Borne Flaviviruses in Saltwater Crocodiles (Crocodylus porosus) in Northern Australia
Source: Viruses. 2022 May 21;14(5):1106. doi: 10.3390/v14051106 (PMC9144604; doi:10.3390/v14051106)
Supplement: Supplementary file 1 [file viruses-14-01106-s001.zip › Table S2. Frequency of co-infection or cross-reactivity 26-04-22.pdf]

Frequency of co-infection or cross-reactivity among tested samples

Farms	WNV+FRV	WNV+SEPV	WNV+STRATV	MVEV+FRV	FRV+STRATV	ALFV+FRV	STRATV+SEPV	FRV+EHV	FRV+SEPV	WNV+STRATV+FRV	WNV+STRATV+SEPV	WNV+STRATV+NMV	FRV+STRATV+SEPV	ALF+FRV+STRATV	MVEV+FRV+STRATV
Overall	12.3	0.8	2.3	0.8	2.3	0.8	0.8	1.5	1.5	5.4	1.5	0.8	0.8	0.8	0.8
Farm A	10.4	1.5	4.5	1.5	4.5	1.5	1.5	3	1.5	10.5	3	1.5	1.5	1.5	1.5
Farm B	14.6	0	0	0	0	0	0	0	2.4	0	0	0	0	0	0
Farm C	13.6	0	0	0	0	0	0	0	0	0	0	0	0	0	0
